# Detailed Analysis of the Human Mitochondrial Contact Site Complex Indicate a Hierarchy of Subunits

**DOI:** 10.1371/journal.pone.0120213

**Published:** 2015-03-17

**Authors:** Christine Ott, Eva Dorsch, Martin Fraunholz, Sebastian Straub, Vera Kozjak-Pavlovic

**Affiliations:** Biocenter, Chair of Microbiology, University of Würzburg, Am Hubland, Würzburg, Germany; Auburn University, UNITED STATES

## Abstract

Mitochondrial inner membrane folds into cristae, which significantly increase its surface and are important for mitochondrial function. The stability of cristae depends on the mitochondrial contact site (MICOS) complex. In human mitochondria, the inner membrane MICOS complex interacts with the outer membrane sorting and assembly machinery (SAM) complex, to form the mitochondrial intermembrane space bridging complex (MIB). We have created knockdown cell lines of most of the MICOS and MIB components and have used them to study the importance of the individual subunits for the cristae formation and complex stability. We show that the most important subunits of the MIB complex in human mitochondria are Mic60/Mitofilin, Mic19/CHCHD3 and an outer membrane component Sam50. We provide additional proof that ApoO indeed is a subunit of the MICOS and MIB complexes and propose the name Mic23 for this protein. According to our results, Mic25/CHCHD6, Mic27/ApoOL and Mic23/ApoO appear to be periphery subunits of the MICOS complex, because their depletion does not affect cristae morphology or stability of other components.

## Introduction

Mitochondria are organelles crucial for a number of cellular functions, such as production of energy by oxidative phosphorylation, cellular metabolism, signaling and apoptosis [1]. Reflecting their endosymbiotic origin, mitochondria possess two membranes that divide them into four subcompartments, outer membrane (OMM), intermembrane space (IMS), inner membrane (IMM) and matrix. The majority of mitochondrial proteins are nuclear-encoded, produced on cytosolic ribosomes and imported into mitochondria with the help of import machineries in mitochondrial membranes.

The OMM of mitochondria hosts the translocase of the outer membrane (TOM) complex, which is the main entry point for the majority of mitochondrial proteins. Depending on the type of the targeting signal, the preproteins are then directed either to the IMM translocases TIM23 and TIM22 complex, or are imported into the IMS by a third route that depends on the mitochondrial intermembrane space assembly machinery (MIA). Finally, the sorting and assembly machinery (SAM/TOB complex) in the OMM directs the membrane integration and assembly of a special group of mitochondrial proteins that are characterized by a β-barrel structure [2]. The SAM/TOB complex is an evolutionary conserved machinery [3–5]. In mammalian mitochondria, it consists of Sam50 and two other subunits, Metaxin 1 and Metaxin 2 [6–9].

The IMM forms invaginations called cristae, which significantly increase its surface and are the site where oxidative phosphorylation takes place. Cristae membrane attaches to the inner boundary membrane at the so-called cristae junction. Cristae formation and stability have been shown to depend on an abundant mitochondrial protein existing in two isoforms of 88 and 90 kDa called Mitofilin [10].

Work performed both in human and yeast mitochondria, identified a Mitofilin-containing protein complex in the IMM as the major organizer of cristae structure. This protein complex has been termed MINOS, for mitochondrial inner membrane organizing system [11], MitOS, for mitochondrial organizing structure [12] or MICOS, for mitochondrial contact site [13]. Recently, a unifying nomenclature has been proposed, where the complex has been assigned the name MICOS, and it components carry names Mic10 to Mic60 [14]. The components of the MICOS complex seem to associate with a number of OMM proteins in yeast mitochondria—the TOM complex [11], Ugo1 and VDAC [12,13] and a fraction of the SAM/TOB complex [13]. In human mitochondria, Mic60/Mitofilin has been connected with several other proteins such as Coiled-coil helix coiled-coil helix domain-containing protein 3 and 6 (Mic19/CHCHD3 and Mic25/CHCHD6), Sam50, Metaxin 1 and 2 and DnaJC11 [15–17]. The interaction of Mic19/CHCHD3 with Mic60/Mitofilin and OPA1 has been proposed to be of importance for the cristae morphology [15]. On the other hand, the interaction of the SAM in the OMM with the MICOS complex appears to be absolutely required for the maintenance of cristae in human mitochondria. Sam50 is found together with MICOS components Mic60/Mitofilin and Mic19/CHCHD3 in the same high molecular weight complex of >1MDa, which was termed mitochondrial intermembrane space bridging (MIB) complex, due to the fact that its components belong both to the IMM and OMM [17]. Recently, other putative components of the human MICOS complex have been characterized in more detail, such as Mic25/CHCHD6 and Mic10/MINOS1 [18,19]. Yet another protein involved in cristae maintenance, MOMA1, has been reported in *Caenorabditis elegans* [20]. Its human homologues are apolipoprotein O (ApoO) and ApoO like (ApoOL) proteins. For the Mic27/ApoOL, a report exists confirming it to be a component of the MICOS complex [21]. ApoO has also been shown to localize to mitochondria [22] and proposed to be a component of the human MICOS complex [21], but this has not yet been fully confirmed.

In this report, using knockdown cell lines, we show detailed analysis of the human MICOS and MIB complex, in respect to the relationship of the individual components with one another. The analysis includes all so far described subunits, except Mic10/MINOS1. In addition, we show that ApoO is detected in the >1 MDa MIB complex after radiolabelled import into mitochondria and co-immunoprecipitates with Mic60/Mitofilin. We, therefore, conclude that ApoO is a novel component of the MICOS complex and propose the name Mic23 for this protein.

## Results

### Expression of Sam50 and Mic19/CHCHD3 leads to the loss of mitochondrial membrane potential

**Fig 1:**
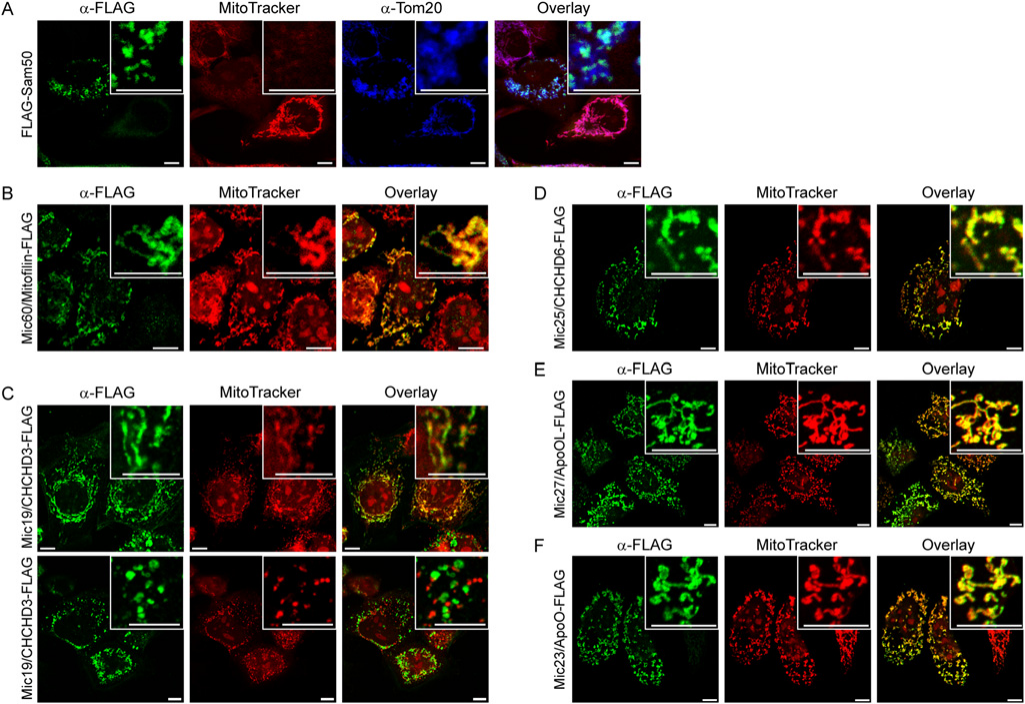
Expression of FLAG-tagged components of the MIB and MICOS complexes. (A-F) HeLa cells were grown on cover slips and transfected using Lipofectamine 2000 with pCDNA3 plasmids carrying information for FLAG-tagged Sam50 (A), Mic60/Mitofilin (B), Mic19/CHCHD3 (C), Mic25/CHCHD6 (D), Mic27/ApoOL (E) or Mic23/ApoO (F). Cells were then labeled with MitoTracker (red channel), fixed and immunostained stained using antibodies directed against the FLAG-tag (green channel) or Tom20 (A, blue channel) and corresponding, fluorophore-coupled secondary antibodes. In the upper right corner enlarged sections can be seen. Scale bar represents 10 μm.

We have cloned Sam50, Mic60/Mitofilin, Mic19/CHCHD3, Mic25/CHCHD6, Mic27/ApoOL and Mic23/ApoO into a mammalian expression vector pCDNA3. The proteins were fused with the FLAG-tag at the carboxy (C)-terminus, except for the Sam50, where the FLAG-tag was located at the amino (N)-terminus, so as not to interfere with the β-sorting signal [23]. The plasmids were then transfected into HeLa cells and analyzed by immunofluorescence. We observed that all the proteins, including Mic23/ApoO-FLAG (Fig. 1F), localized to mitochondria upon expression (Fig. 1). In the case of Sam50, already observed mitochondrial fragmentation and loss of membrane potential occurred [23], probably due to the accumulation of the non-assembled Sam50 in the IMS (Fig. 1A). Interestingly, we observed the similar effect of Mic19/CHCHD3-FLAG expression on mitochondria in many (Fig. 1C, lower panel), but not all cells (Fig. 1C, upper panel). Mitochondria in approximately 50% of cells expressing Mic19/CHCHD3-FLAG lost membrane potential (Fig. 1C, lower panel) and were significantly shorter than the mitochondria from non-transfected cells, or from the Mic19/CHCHD3-FLAG-expressing cells where mitochondrial membrane potential was unaffected (S1 Fig.). We conclude that Mic23/ApoO is a mitochondrial protein and that expression of Sam50 and Mic19/CHCHD3 has a deleterious effect on mitochondrial membrane potential.

### The key components of the MICOS and MIB complex are Mic60/Mitofilin, Mic19/CHCHD3 and Sam50

**Fig 2:**
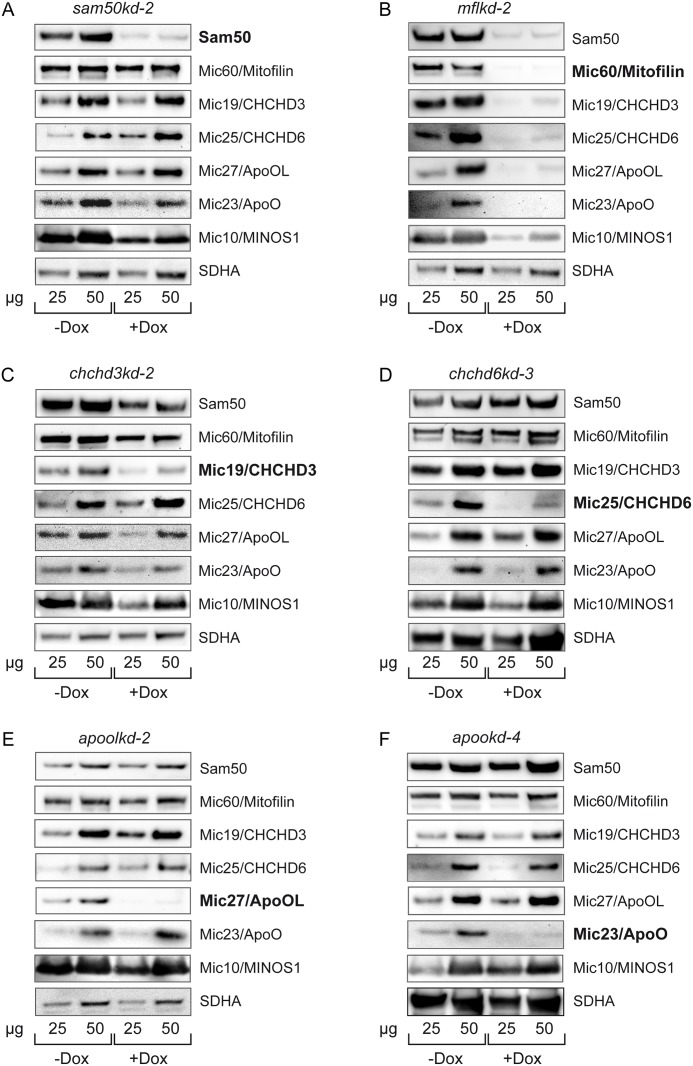
Steady state levels of MIB and MICOS components in knockdown cell lines. (A-F) Inducible knockdown cell lines, carrying shRNA that downregulates Sam50 (A, *sam50kd-2*), Mic60/Mitofilin (B, *mflkd-2*), Mic19/CHCHD3 (C, *chchd3kd-2*), Mic25/CHCHD6 (D, *chchd6kd-3*), Mic27/ApoOL (E, *apoolkd-2*) or Mic23/ApoO (F, *apookd-4*) were generated. Cells were induced with doxycycline (Dox) for 7 days and mitochondria were isolated from non-induced (-Dox) and induced cells (+Dox). 25 and 50 μg of mitochondrial protein from-Dox and +Dox samples were analyzed by SDS-PAGE and western blot, using antibodies directed against Sam50, Mic60/Mitofilin, Mic19/CHCHD3, Mic25/CHCHD6, Mic27/ApoOL, Mic23/ApoO, Mic10/MINOS1 and SDHA. SDHA—succinate dehydrogenase, a component of the mitochondrial respiratory complex II.

To examine the interdependence of the human MICOS and MIB components, we created knockdown cell lines of Mic25/CHCHD6 (*chchd6kd-3*), Mic27/ApoOL (*apoolkd-2*) and Mic23/ApoO (*apookd-4*), in addition to already described knockdown cell lines of Sam50 (*sam50kd-*2), Mic60/Mitofilin (*mflkd-2*) and Mic19/CHCHD3 (*chchd3kd-2*) [17]. We induced the knockdown of the respective component of the MICOS and the MIB complex for 7 days. In our experience, 7 days is the time point at which the effects of the knockdown on other complex components become visible, whereas side-effects are kept to the minimum. In all of the cell lines except *chchd3kd-*2 the knockdown of the targeted protein was very efficient (approximately >90%) (Fig. 2). In *chchd3kd-*2 we estimated that the knockdown efficiency was in the range of 70–80% (Fig. 2C). We have observed before that the knockdown of one component of the protein complex can adversely affect the amounts of other components. This depends, however, on the importance of the downregulated protein for the overall stability of the complex [9,17]. Here we observed that the greatest effect on the MICOS and MIB complex had the knockdown of Mic60/Mitofilin. The depletion of Mic60/Mitofilin led to a complete loss of the OMM protein Sam50, the MIB complex component, as reported [17], and it also dramatically affected MICOS components Mic19/CHCHD3, Mic25/CHCHD6, Mic27/ApoOL, Mic10/MINOS1, as well as Mic23/ApoO, confirming that there is a functional relationship between Mic60/Mitofilin and Mic23/ApoO. As a control, levels of the respiratory chain complex II protein SDHA remained unchanged (Fig. 2B). Another component of importance for the stability of the MICOS and MIB complex is Mic19/CHCHD3. The depletion of this protein led to reduction in Sam50, Mic60/Mitofilin, Mic27/ApoOL, Mic10/MINOS1 and Mic23/ApoO levels, whereas the amount of Mic25/CHCHD6 was not affected (Fig. 2C). The depletion of Mic25/CHCHD6, Mic27/ApoOL and Mic23/ApoO had no effect on other MICOS components or Sam50 (Fig. 2D-F). As reported, the knockdown of Sam50 did not affect the levels of Mic60/Mitofilin, whereas Mic19/CHCHD3 levels were only mildly reduced [17]. Mic25/CHCHD6 and Mic27/ApoOL remained undisturbed by Sam50 depletion, but we observed reduction in Mic10/MINOS1 levels and a mild reduction in the Mic23/ApoO levels following Sam50 knockdown (Fig. 2A).

**Fig 3:**
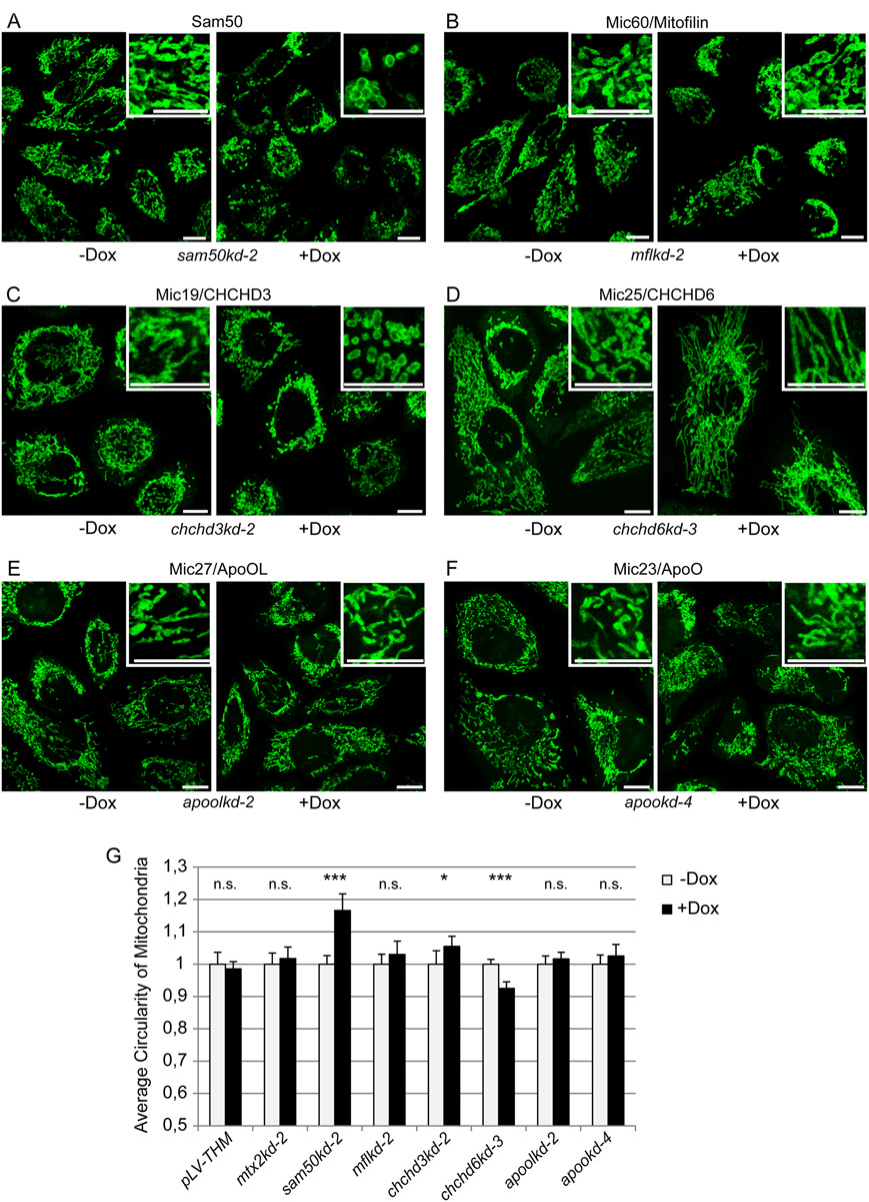
Mitochondrial morphology in knockdown cell lines. (A-F) Cells from inducible knockdown cell lines, carrying shRNA that downregulates Sam50 (A, *sam50kd-2*), Mic60/Mitofilin (B, *mflkd-2*), Mic19/CHCHD3 (C, *chchd3kd-2*), Mic25/CHCHD6 (D, *chchd6kd-3*), Mic27/ApoOL (E, *apoolkd-2*) or Mic23/ApoO (F, *apookd-4*) were grown on coverslips and induced with doxycycline (Dox) for 7 days. After fixation, mitochondria were decorated with anti-Tom20 antibody and Cy5-coupled secondary antibody. Cells were analyzed by fluorescence microscopy. Enlarged sections of the shown pictures are represented in the upper right corner. Scale bar represents 10 μm. (G) Average circularity as a measurement of mitochondrial fragmentation was analyzed for 10 fields of view per sample (~200 cells) using Image J. *pLV-THM*, an empty vector cell line, and a *mtx2kd-2* cell line carrying shRNA against Metaxin 2 served as controls (S2 Fig.). Significance was established using student’s t-test on the average circularity data. Data were normalized by setting the non-induced (-Dox) values to 1. * p<0.05, *** p<0.0001, n.s. not significant.

The same knockdown cell lines were used for the analysis of mitochondrial network and cristae structure in the mitochondria of cells expressing respective shRNAs. As reported, knockdown of Sam50 leads to a significant fragmentation of mitochondria (Fig. 3A and G) [17]. Knockdown of Mic19/CHCHD3 caused mitochondrial fragmentation already after 7 days of knockdown induction (Fig. 3C and G). This effect increased after the induction of the knockdown was prolonged to 14 days, whereas empty vector control cell line, and a control cell line where Metaxin 2 was downregulated did not show similar phenotype (S2 C-F Fig.). Surprisingly, the knockdown of Mic60/Mitofilin did not cause significant fragmentation of mitochondria (Fig. 3B and G), whereas the knockdown of Mic25/CHCHD6 caused mitochondrial elongation (Fig. 3D and G). The knockdown of Mic27/ApoOL and Mic23/ApoO had no significant effect on mitochondrial morphology (Fig. 3E-G). As controls, mitochondrial morphology was assessed in a 7 days induced empty vector cell line *pLV-THM* and a *mtx2kd-2* cell line expressing an shRNA directed against Metaxin 2 (S2 Fig., Fig. 3G), which has already been shown to have no effect on mitochondrial cristae morphology [17].

**Fig 4:**
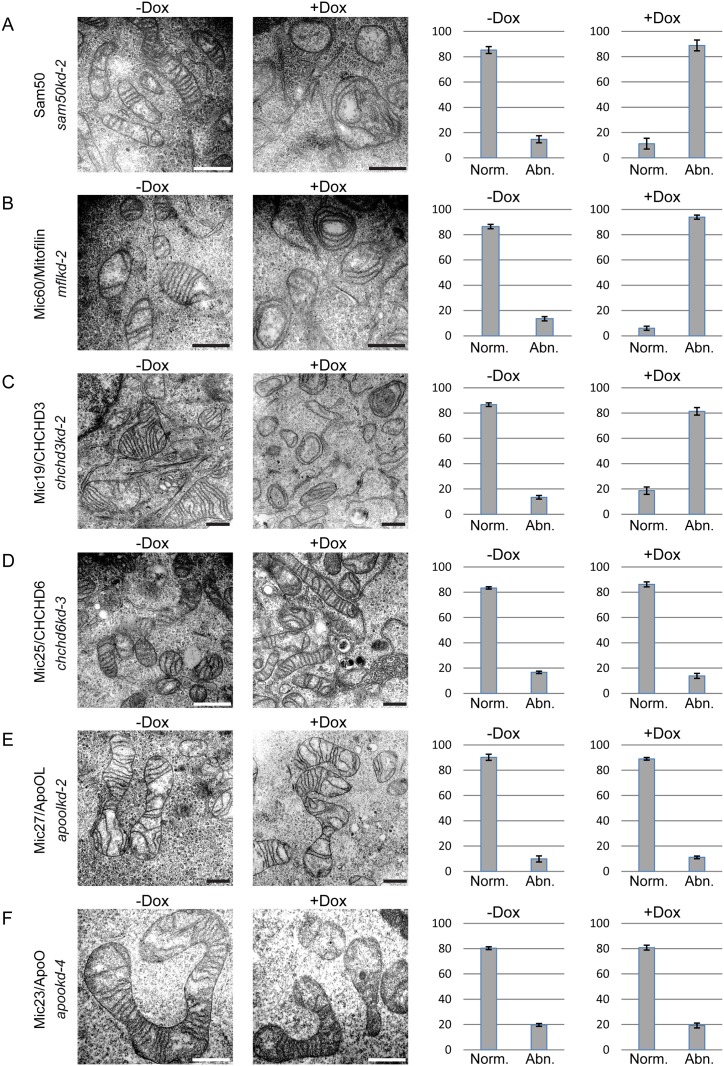
Transmission electron microscopy of MIB and MICOS components knockdown cell lines. (A-F) Inducible knockdown cells as described in Fig. 2, carrying shRNA that downregulates Sam50 (A, *sam50kd-2*), Mic60/Mitofilin (B, *mflkd-2*), Mic19/CHCHD3 (C, *chchd3kd-2*), Mic25/CHCHD6 (D, *chchd6kd-3*), Mic27/ApoOL (E, *apoolkd-2*) or Mic23/ApoO (F, *apookd-4*) were seeded on cover slips, fixed and analyzed using transmission electron microscopy. All cells were induced with doxycycline (Dox) for 7 days, except *chchd3kd-*2, which was induced for 14 days. Scale bar represents 500 nm. The graphs on the right hand side represent mean values of at least hundred mitochondria counted from two different regions of two different samples ± SD. The mitochondria were divided into two groups, abnormal (Abn.) with circular stacks of inner membrane that appear instead of usual cristae and normal (Norm.) where the usual parallel cristae folds are visible.

Regarding the structure of cristae, we observed the complete loss of mitochondrial cristae and appearance of mitochondria with concentric, onion-like inner membrane in cells where Sam50, Mic60/Mitofilin and Mic19/CHCHD3 were depleted (Fig. 4A-C), as reported and expected [10,15,17]. Of note, Mic19/CHCHD3 knockdown had to be induced for 14 days for this analysis, instead of the usual 7 days, because at the earlier time point the cristae phenotype was not sufficiently pronounced. Interestingly and contrary to the recent reports [18,21], knockdown of Mic25/CHCHD6 or of Mic27/ApoOL had no visible effect on cristae morphology (Fig. 4D and E). This did not change even after prolonged knockdown induction for 14 days (data not shown). In line with these observations, knockdown of Mic23/ApoO also had no effect on mitochondrial cristae structure (Fig. 4F). We conclude that Mic60/Mitofilin, Mic19/CHCHD3 and Sam50 are vital components of the MICOS and MIB complexes, whereas Mic25/CHCHD6, Mic27/ApoOL and Mic23/ApoO appear to be periphery subunits, with little or no role in maintenance of cristae stability.

### Mic23/ApoO is a subunit of the MICOS complex

**Fig 5:**
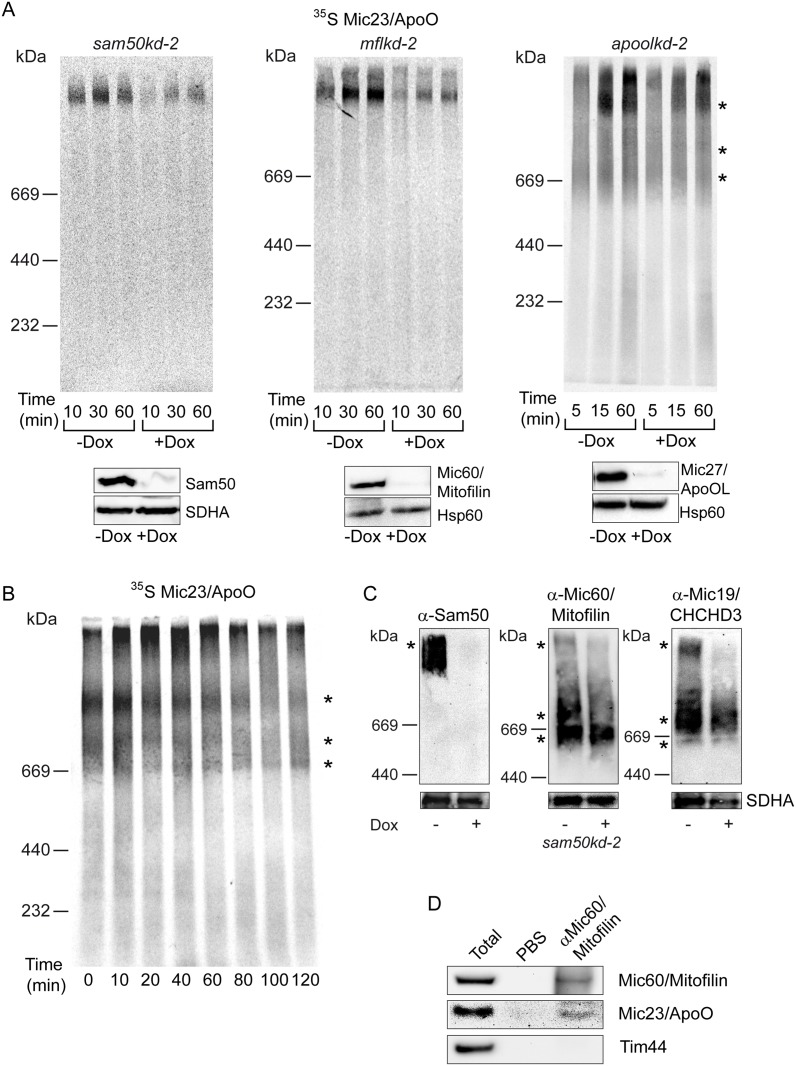
Mic23/ApoO is co-precipitated with Mic60/Mitofilin and its import is affected by depletion of Mic60/Mitofilin and Sam50. (A) Knockdown of Mic60/Mitofilin, Sam50 and Mic27/ApoOL was induced for 7 days using doxycycline (Dox) in the respective knockdown cell line (*mflkd-2* for Mic60/Mitofilin, *sam50kd-2* for Sam50 and *apoolkd-2* for Mic27/ApoOL). Mitochondria were isolated and 50 μg of mitochondria per lane was incubated with ^35^S-labeled Mic23/ApoO for indicated times. Lower panels represent western blot controls of the knockdown and loading, using antibodies against Mic60/Mitofilin, Sam50 or Mic27/ApoOL and Hsp60 or SDHA. Hsp60—heat shock protein 60, SDHA—succinate dehydrogenase, a component of the mitochondrial respiratory complex II. (B) Pulse-chase experiment was performed with 50 μg of HeLa mitochondria per lane, which were incubated with ^35^S-labeled Mic23/ApoO for 20 min to allow formation of the Mic23/ApoO import complex. Mitochondria were reisolated, incubated in the import buffer for indicated times and analyzed by BN-PAGE and autoradiography. (C) Inducible knockdown cell line carrying shRNA that downregulates Sam50 (*sam50kd-2*) was induced for 7 days with doxycycline (Dox) and mitochondria were isolated from non-induced (-Dox) and induced cells (+Dox). 50 μg of mitochondrial protein per lane was solubilized in 1% digitonin buffer and analyzed by blue native (BN)-PAGE and western blot, using antibodies directed against Sam50, Mic60/Mitofilin or Mic19/CHCHD3, and, as control, SDHA (lower panels). SDHA—succinate dehydrogenase, a component of the mitochondrial respiratory complex II. Asterisks indicate three protein complexes observed after ^35^S-labeled Mic23/ApoO import or western blot analysis of the steady state levels of Sam50, Mic60/Mitofilin and Mic19/CHCHD3. (D) Co-immunoprecipitation was performed by targeting Mic60/Mitofilin with specific antibodies and analyzing the precipitates by SDS-PAGE and western blot, using Mic60/Mitofilin, Mic23/ApoO and Tim44 antibodies. PBS was used as a negative control. Tim44—translocase of the inner mitochondrial membrane 44.

To further address Mic23/ApoO as a potential novel subunit of the MICOS complex, we have developed an in vitro import assay into isolated mitochondria for the radiolabelled precursor of Mic23/ApoO (Fig. 5A). After import, the precursor could be detected in a >1 MDa large protein complex (Fig. 5A, asterisk). The quantity of this complex increased in a time-dependent manner, but the formation of the complex was severely affected by the depletion of either Sam50 or Mic60/Mitofilin. The depletion of Mic27/ApoOL, on the other hand, affected the formation of the complex only mildly and at earlier time points (Fig. 5A). The lower panels show western blot controls of the Sam50, Mic60/Mitofilin or Mic27/ApoOL knockdown and of the mitochondrial amounts used in the experiment. We also observed the appearance of two lower molecular weight complexes after the import of Mic23/ApoO into the mitochondria of *apoolkd-2* cells (Fig. 5A, asterisks). To further study the dynamics of the assembly of the precursor of Mic23/ApoO, we performed pulse-chase experiment into isolated HeLa mitochondria. With these mitochondria, two lower molecular weight complexes were even more pronounced (Fig. 5B, asterisks). With time, the amount of radiolabeled Mic23/ApoO in the high molecular weight complex diminished. However, at the same time the intensity of the two lower molecular weight complexes only mildly increased (Fig. 5B). The high molecular weight complex of Mic23/ApoO resembles the one observed after the import of the radiolabeled precursor of Mic19/CHCHD3 [17] and corresponds in size to the MIB complex detected after BN-PAGE analysis of the steady state levels of Sam50 in *sam50kd-2* mitochondria (Fig. 5C, left hand panel, asterisk). Two lower molecular weight complexes could represent the MICOS complex, as they resemble two lower complexes detected after BN-PAGE/western blot analysis of Mic60/Mitofilin and Mic19/CHCHD3 in *sam50kd-2* mitochondria (Fig. 5C, middle and right hand panel, asterisks). Finally, we performed a co-immunoprecipitation by targeting Mic60/Mitofilin with specific antibodies. We observed that Mic23/ApoO co-precipitated with Mic60/Mitofilin, whereas a control protein Tim44 did not (Fig. 5D). These results confirm Mic23/ApoO as a subunit of the MIB and MICOS complex, and show that newly acquired precursor of Mic23/ApoO is found in the large MIB complex in the association with Sam50.

## Discussion

In this work, we have analyzed in detail the relationship between most of the described components of the human MICOS complex. In addition, we have included Sam50 in the analysis, the subunit of the SAM complex, which together with the MICOS complex assists in mitochondrial cristae formation and forms the mitochondrial bridging MIB complex. The only described MICOS subunit we have not addressed is Mic10/MINOS1, due to the fact that we were not able to create satisfactory knockdown cell line for this protein. For other proteins, we have used knockdown cells lines where each of the components was downregulated and could observe how individual proteins of the MICOS and MIB complexes affect other subunits. We have also shown that the suspected subunit apolipoprotein O is indeed a part of the MICOS complex and we assign the name Mic23 to it.

The knockdown cell lines we used are a valuable and reliable tool to study protein function. As we described in our previous publications and supported with various analysis, including analyzing mitochondrial morphology and protein levels in a control, empty vector cell line *pLV-THM* [9,17,24], the addition of 1 μg/ml doxycycline to the growth medium does not affect mitochondrial function overall, but is sufficient to reproducibly induce shRNA-mediated knockdown of specific proteins. Also, the knockdown of the targeted protein becomes significant only after 4–5 days of the induction with doxycycline [17], meaning that the time point of 7 days induction that we use for our analysis is the optimal time where we see the effect of the knockdown with minimal side effects. Even the prolonged induction of the knockdown for up to 28 days of doxycycline treatment affects significantly only the targeted protein and proteins that are functionally connected to it, whereas most of the other mitochondrial proteins remain unaffected [17]. We believe, therefore, that the analysis we performed on the described knockdown cell lines reflect reliably the effects that the depletion of the targeted protein has on mitochondria.

We have expressed FLAG-tagged subunits of the MICOS complex, as well as Sam50, and could see that all proteins localize to mitochondria only, including Mic23/ApoO, which agrees with recent reports [21,22]. As described, we observed deleterious effect of Sam50 expression on mitochondrial integrity and membrane potential (Fig. 1A), which could be explained by the accumulation of this β-barrel protein in the IMS [23]. Interestingly, we observed mitochondrial fragmentation and loss of membrane potential in approximately 50% of the cells expressing Mic19/CHCHD3, as well (Fig. 1C, lower panel, S1 Fig.). It is possible that large amounts of this protein produced from the plasmid cannot be efficiently integrated into the MICOS complex and also accumulate in the IMS, which is sensitive to protein aggregation [23].

Our further observations of protein levels in the cell lines where individual components of the MICOS and MIB complexes have been depleted show that the central subunit of the MICOS and the MIB complex is Mic60/Mitofilin. Depletion of this IMM protein leads to almost complete loss of all other MICOS components we tested, as well as Sam50 (Fig. 2B). Mic19/CHCHD3 also plays an important role in the MICOS complex organization, because its depletion affects the levels of Mic60/Mitofilin and Sam50, whereas Mic27/ApoOL, Mic10/MINOS1 and Mic23/ApoO are also affected, although to a lesser extent (Fig. 2B). The third in the hierarchy of subunits is an OMM protein Sam50. Interestingly, its depletion does not in the least affect levels of Mic60/Mitofilin (Fig. 2A, [17]), but leads to a slight reduction in Mic19/CHCHD3 and Mic23/ApoO levels and affects significantly Mic10/MINOS1 (Fig. 2A). This underlines the possible interaction between Sam50 and Mic19/CHCHD3, as reported by Darshi and colleagues [15]. Other three MICOS components we tested, Mic25/CHCHD6, Mic27/ApoOL and Mic23/ApoO appear to be periphery components, since their deletion does not affect any remaining MICOS or MIB component (Fig. 2D-F).

The conclusions about the hierarchy of subunits are further confirmed by the analyses of mitochondrial network and electron microscopy analyses of the mitochondria from the knockdown cell lines. Only the knockdown of Sam50, Mic60/Mitofilin and Mic19/CHCHD3 affects the morphology of mitochondrial cristae (Fig. 3A-C), as reported [10,15,17]. Mitochondrial fragmentation is also present in Sam50 and Mic19/CHCHD3 knockdown cells (Fig. 3A, C, G). However, the fragmentation of Mic60/Mitofilin knockdown mitochondria is not significantly pronounced, according to the quantification we performed (Fig. 3B and G). Even though this could be a consequence of the limited sensibility of the quantification method we used, the quantified effect corresponds to what we see on the pictures in all cases (Fig. 3 and 4). The effect of Mic19/CHCHD3 knockdown on cristae stability was evident only after extended depletion of 14 days instead of usual 7 days (Fig. 4C), even though the fragmentation of mitochondria was visible after 7 days of knockdown (Fig. 3C), but increased after 14 days (S2E and F Fig.). This might be due to the lower efficiency of the knockdown as compared to other cell lines. The remaining Mic19/CHCHD3 protein could still perform its function up to a certain extent and only a prolonged depletion of this MICOS subunit would then lead to cristae instability. The knockdown of the periphery subunits Mic25/CHCHD6, Mic27/ApoOL and Mic23/ApoO does not affect cristae morphology, which corresponds to the lack of any effect that these proteins have on other MICOS subunits (Fig. 2 and Fig. 4 D-F). This, however, contrasts previous reports, where Mic25/CHCHD6 and Mic27/ApoOL were found to be required for cristae stability [18,21]. We would nevertheless like to point out that our system using single cell clones of knockdown cell lines enables us to study large amount of cells with a uniform knockdown. In the case of both Mic25/CHCHD6 and Mic27/ApoOL the great majority of the cells lacking the respective protein showed no alterations in cristae morphology (Fig. 3). This did not change even after prolonged depletion of 14 days (data not shown). Therefore, we find it highly likely that these two proteins indeed represent periphery subunits, with no major role in cristae stabilization. This would be in agreement with the findings in yeast mitochondria, where the deletion of different MICOS components had strong to intermediate effects on cristae morphology, with some of them having no effect at all, and where the absence of Mic27, ApoOL homolog, only moderately affected cristae shape [11]. Interestingly, we observed significant elongation of mitochondria after the knockdown of Mic25/CHCHD6 (Fig. 3D and G). Whether this is an effect of the Mic25/CHCHD6 absence or the characteristic of the cell clone we used needs to be clarified in the future.

We have also established an in vitro import of Mic23/ApoO into isolated mitochondria. The protein assembles in a time-dependent manner into a protein complex of a size similar to the complex we observe after the import of Mic19/CHCHD3 (Fig. 5A, [17]). Also, the amount of this complex is significantly affected by the depletion of either Sam50 or Mic60/Mitofilin (Fig. 5). This is not due to the import defect, because we have already shown that Sam50-depleted mitochondria generally efficiently import other mitochondrial precursors [17]. Together with the data from the Fig. 2 and co-immunoprecipitation data from the Fig. 5 this supports the idea of Mic23/ApoO being the part of the MICOS and MIB complexes. Of interest, however, is to notice that the proteins of the MICOS complex we imported so far can be detected mostly in the high molecular weight complex corresponding to the MIB complex, and only weakly in the lower molecular weight MICOS complexes we detect in steady state levels (Fig. 5, [17]). This changed, however, when we used mitochondria isolated from HeLa cells, where MICOS complexes after Mic23/ApoO import were more obvious (Fig. 5B). Therefore, the visibility of these complexes upon import might depend on the cell line used. It is possible that newly imported subunits first associate with the large, two membrane spanning MIB complex. The exchange of the endogenous, non-labelled subunits of the MICOS complex with the new, radiolabeled subunits could be a slow process, because even though we see a gradual reduction of the MIB complex signal in our pulse-chase experiment in the course of 120 min, we do not observe the corresponding increase in the signal of the MICOS complexes (Fig. 5B).

In conclusion, our work emphasizes once more the importance of the communication between the IMM MICOS and the OMM SAM complex, which exist in the one membrane bridging MIB complex. We also confirm Mic23/ApoO as a new subunit of the MICOS complex, and establish a hierarchy of subunits pointing to Mic60/Mitofilin, Mic19/CHCHD3 and Sam50 as being the central components of the MICOS and the MIB complex and Mic25/CHCHD6, Mic27/ApoOL and Mic23/ApoO as having a peripheral role. In future, it would be interesting to analyze in detail Mic10/MINOS1 in relation to other MICOS components. We can only presume, according to the available data [19], that Mic10/MINOS1 would also be one of the key components of the MICOS complex. Also, better understanding of the association of DnaJC11 with the MICOS or the MIB complex should be one of the future tasks.

## Materials and Methods

### Cell culture and isolation of mitochondria

Inducible, shRNA-mediated knockdown HeLa cell lines were generated as described previously [9,25]. The sequence of *sam50kd-2*, *mflkd-2* and *chchd3kd-2* were published before [9,17]. The sequence of *chchd6kd-*3 shRNA is 5’-GGGTGTCAAGAGGTATGAACA-3’, *apoolkd-*2 is 5’-GCAAGGGTGTTTATGTCTTTG-3‘ and of *apookd-4* is 5’-GGTTTACGAGGATATATAGTC-3’. Single cell clones were isolated for each shRNA, and tested by western blot. Cells were cultivated in RPMI 1640 or DMEM (Gibco) supplemented with 10% FCS (Biochrom) and penicillin/streptomycin. shRNA expression was induced by adding 1 μg/ml doxycycline (BD Biosciences) to the growth medium for indicated time periods, mostly 7 days. Isolation of mitochondria was performed as already described [9].

### Microscopy

Transmission electron microscopy was performed as described [17]. For the fluorescence microscopy, cells grown on coverslips were stained using 150 nM MitoTracker (Molecular Probes) in cell culture medium for 30 min at 37°C. Samples were washed with PBS, fixed in 3.7% PFA and decorated with antibodies against FLAG-tag and Tom20, and the secondary antibodies coupled to fluorophores. After mounting of the coverslips onto microscopy slides, samples were analyzed using a Leica TCS SPE confocal microscope. Mitochondrial length was assessed using Image J and the data was normalized by setting the value for non-transfected cells to 1.

For quantification of mitochondrial fragmentation, mitochondria from non-induced and doxycycline-induced samples were seeded on coverslips 2 days prior the fixation and labeled using an anti-Tom20 antibody and a Cy5-labeled anti-mouse secondary antibody. Imaging was performed on a Leica TCS SP5 confocal laser scanning microscope using the 63x/1.4 NA oil immersion objective with the 633 nm laser line for excitation of the fluorophore and a 643–743 nm bandpass for emission detection. Gain settings and laser intensities were identical for recording of all the samples for the subsequent quantification of mitochondrial fragmentation parameters. 10 fields of view were recorded per sample (164x164 microns, 2048x2048 pixels, 8 bit depth), thus representing an area of roughly 500 μm^2^ and approximately 200 cells. Fiji/ImageJ (www.fiji.sc) was used to determine the circularity of Tom20-stained mitochondria, similar to the method described in Dagda et al. [26]. Background was subtracted and a median filter was applied (radius 1.5). For detection of regions of interest, the contrast was enhanced (0.8% saturated pixels) and automated OTSU thresholding was performed. The circularity of the identified ROIs was determined by “Analyze particles” and the average circularity was determined for each of the 10 slices per sample. Data were normalized by setting the non-induced (-Dox) values to 1. Significance was established using student’s t-test on the average circularity data.

### Protein import, electrophoresis, western blot and immunoprecipitation

Transcription and translation were performed in the presence of ^35^S-methionine/cysteine (PerkinElmer) using TnT SP6 Quick Coupled System (Promega). Freshly isolated mitochondria from induced and non-induced knockdown cell lines were incubated at 37°C with the radiolabeled proteins in the import buffer (250 mM sucrose, 20 mM Hepes, 80 mM KCl, 5 mM MgCl_2_, 3% BSA (w/v), 2 mM KH_2_PO_4_, 5 mM methionine, 10 mM Na-succinate, 2 mM ATP) for the indicated time periods. For the pulse-chase experiment, mitochondria were incubated with the radiolabeled protein for 20 min at 37°C, reisolated and resuspended in the fresh import buffer. Mitochondria were then further incubated at 37°C and samples were taken at 0, 10, 20, 40, 60, 80 and 120 min. Samples were lysed in 1% digitonin buffer (1% digitonin (Sigma) in 20 mM Tris—HCl, 0.1 mM EDTA, 50 mM NaCl, 10% (v/v) glycerol, pH 7.4), mixed with 5 ml of loading dye (5% (w/v) Coomassie brilliant blue G-250, 150 mM Bis-Tris, 500 mM ε-amino-n-caproic acid, pH 7.0) and analyzed on 4–10% gels by BN-PAGE [27]. ^35^S-labeled proteins were visualized by autoradiography using a Typhoon imaging system (GE Healthcare). For western blot analysis, mitochondria were solubilized in 1% digitonin buffer and separated using 4–10% BN-gels. Gels were blotted onto a polyvinylidene fluoride (PVDF) membrane, blocked with 5% milk in TBS buffer (50 mM Tris-HCl, pH 7.6, 150 mM NaCl) and decorated with indicated antibodies. Co-immunoprecipitation was performed as described [28].

### Antibodies

ApoO and ApoOL antibodies were purchased from SIGMA, Tom20 from BD Biosciences, Mitofilin, CHCHD3 and CHCHD6 from Abcam, SDHA from Invitrogen, MINOS1 from GeneTex and Hsp60 from Stressgen. Sam50 antibody was raised in rabbits against a full-length 10xHis-tagged protein. Secondary anti-rabbit and anti-mouse antibodies coupled to Cy3 or Cy5 fluorophores were purchased from Dianova.

## Supporting Information

S1 Fig Expression of FLAG-tagged Mic19/CHCHD3 causes mitochondrial shortening. HeLa cells were grown on cover slips and transfected with pCDNA3 plasmids carrying information for FLAG-tagged Mic19/CHCHD3 as described in Fig. 1. Cells were labeled with MitoTracker, fixed and immunostained using antibodies directed against the FLAG-tag and fluorophore-coupled secondary antibodes. The average length of mitochondria was determined using Image J from 10 fields of view (~30 cells), accounting for the zoom factor. Cells were divided in three groups, non-transfected, transfected cells where expressed FLAG-Mic19/CHCHD3 did not cause loss of mitochondrial membrane potential as assessed by MitoTracker staining, and transfected cells where the loss of mitochondrial membrane potential occurred. Data were normalized by setting the value for non-transfected cells to 1. The graph represents mean value ± SD.
(TIF)

S2 Fig Mitochondrial morphology in knockdown cell lines in controls and after 14 days of induction. (A,B) *pLV-THM*, an empty vector cell line (A), and an *mtx2kd-2* cell line carrying shRNA against Metaxin 2 (B) were grown on coverslips and induced for 7 days with doxycycline (Dox). After fixation, mitochondria were decorated with anti-Tom20 antibody and Cy5-coupled secondary antibody and analyzed by fluorescence microscopy. Enlarged sections are shown in the upper right corner. Scale bar represents 10 μm. (C-E) Control cell lines as in (A) and (B), as well as an inducible knockdown cell lines, carrying shRNA that downregulates Mic19/CHCHD3 (*chchd3kd-2*) were grown on coverslips and induced for 14 days with doxycycline (Dox). Cells were treated as in (A) and (B) and analyzed by fluorescence microscopy. Enlarged sections are shown in the upper right corner. Scale bar represents 10 μm. (F) Average circularity of mitochondria as a measurement of mitochondrial fragmentation was analyzed for 10 fields of view per sample (~200 cells) using Image J. Significance was established using student’s t-test on the average circularity data. Data were normalized by setting the non-induced (-Dox) values to 1. * p<0.05, *** p<0.0001, n.s. not significant.
(TIF)
