# Supplementary figures and images for: Detailed Analysis of the Human Mitochondrial Contact Site Complex Indicate a Hierarchy of Subunits
Source: PLoS One. 2015 Mar 17;10(3):e0120213. doi: 10.1371/journal.pone.0120213 (PMC4363703; doi:10.1371/journal.pone.0120213)

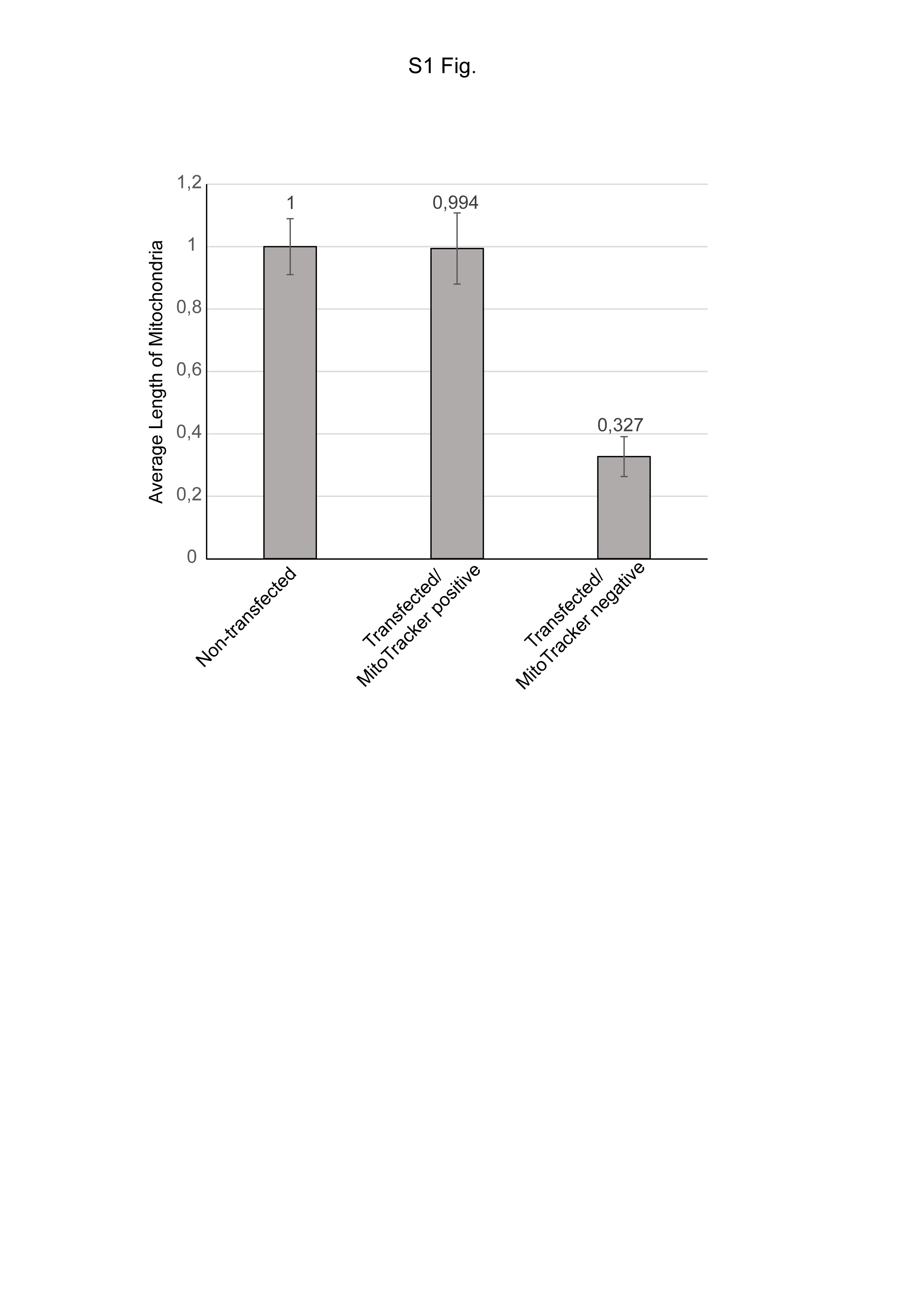

Supplement: S1 Fig — HeLa cells were grown on cover slips and transfected with pCDNA3 plasmids carrying information for FLAG-tagged Mic19/CHCHD3 as described in Fig. 1. Cells were labeled with MitoTracker, fixed and immunostained using antibodies directed against the FLAG-tag and fluorophore-coupled secondary antibodes. The average length of mitochondria was determined using Image J from 10 fields of view (~30 cells), accounting for the zoom factor. Cells were divided in three groups, non-transfected, transfected cells where expressed FLAG-Mic19/CHCHD3 did not cause loss of mitochondrial membrane potential as assessed by MitoTracker staining, and transfected cells where the loss of mitochondrial membrane potential occurred. Data were normalized by setting the value for non-transfected cells to 1. The graph represents mean value ± SD. (TIF) [file pone.0120213.s001.tif]

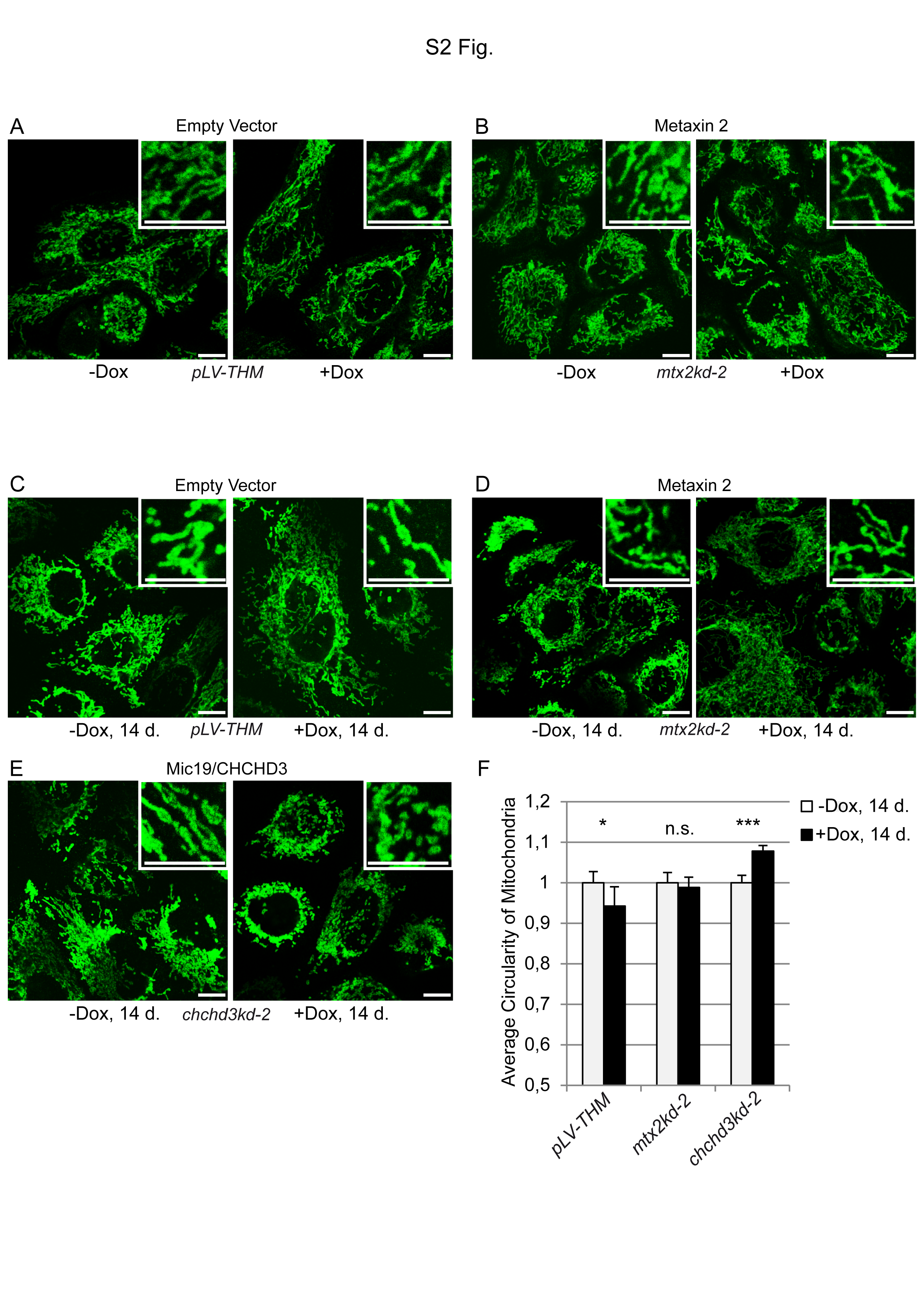

Supplement: S2 Fig — (A,B) pLV-THM, an empty vector cell line (A), and an mtx2kd-2 cell line carrying shRNA against Metaxin 2 (B) were grown on coverslips and induced for 7 days with doxycycline (Dox). After fixation, mitochondria were decorated with anti-Tom20 antibody and Cy5-coupled secondary antibody and analyzed by fluorescence microscopy. Enlarged sections are shown in the upper right corner. Scale bar represents 10 μm. (C-E) Control cell lines as in (A) and (B), as well as an inducible knockdown cell lines, carrying shRNA that downregulates Mic19/CHCHD3 (chchd3kd-2) were grown on coverslips and induced for 14 days with doxycycline (Dox). Cells were treated as in (A) and (B) and analyzed by fluorescence microscopy. Enlarged sections are shown in the upper right corner. Scale bar represents 10 μm. (F) Average circularity of mitochondria as a measurement of mitochondrial fragmentation was analyzed for 10 fields of view per sample (~200 cells) using Image J. Significance was established using student’s t-test on the average circularity data. Data were normalized by setting the non-induced (-Dox) values to 1. * p<0.05, *** p<0.0001, n.s. not significant. (TIF) [file pone.0120213.s002.tif]
